# Retrospective analysis and nursing management of inpatient falls: Case series

**DOI:** 10.1097/MD.0000000000027977

**Published:** 2021-11-24

**Authors:** Xiaoyan Liu, Xiaoling Zhu, Yan Song

**Affiliations:** a Geriatric Department, Affiliated Hospital of Nantong University, Medical School, Nantong University, Nantong, Jiangsu, China; b Clinical Medicine Department, Affiliated Hospital of Nantong University, Nantong, Jiangsu, China; c Medical School, Nantong University, Nantong, Jiangsu, China.

**Keywords:** adverse events, fall, inpatients, nursing management

## Abstract

**Background:**

Falls are common adverse events: approximately 1 million falls occur in hospitals annually, accounting for approximately 70% of inpatient accidents.

**Objectives:**

This study aimed to identify the characteristics of adverse fall events in our hospital from January 2019 to October 2021; it also had the goal of formulating nursing management countermeasures to reduce the occurrence of falls in our hospital. Identify the impact of formulated fall prevention and Group standards.

**Methods:**

Details of falls were obtained from the nursing adverse event reporting system of the Affiliated Hospital of Nantong University from January 2019 to December 2020. We analyzed 76 falls reported to the nursing department using a nonpunitive reporting system. We established fall prevention and Group standards. And compared with falls from March to October 2021.

**Results:**

In the study period, before the intervention, 76 falls occurred in the hospital: 18 in the day shift and 58 in the night shift. Among the falls, 32 (42.1%) occurred at the bedside; that figure was followed by 26 in the toilet (34.2%) and 18 in other places outside the ward (23.7%). The top 3 causes of the falls accounted for 84.2% of the cases: 14 were caused by nurses; 20 were caused by the patients themselves; and 30 cases were caused by concomitant factors. Regarding the consequences of the falls, 18 patients were uninjured, 22 had a mild injury, 12 were moderately injured, and 24 suffered severe injuries. After the intervention, there were fewer falls per patient day and when patients were less likely to be injured in a fall (*P *< .05).

**Conclusions:**

Enhancing awareness of factors that lead to inpatients falls may reduce the risk of concomitant injuries. Group standards should be established to prevent falls. In accordance with standards, it is necessary to consider health education and process supervision; it is also important to encourage inpatients to participate in safety management and to effectively ensure the safety of patients.

## 1 Introduction

A fall is defined as an involuntary change in posture whereby the subject ends up on the floor. It refers to unexpected position changes caused by complex disease conditions, reduced adaptability, or other factors leading to loss of body balance. The result may be a seizure, stroke, loss of consciousness, and susceptibility to external forces owing to the inability to resist.^[1]^ Inpatient falls are an ongoing problem in health-care settings,^[2]^ accounting for 20% to 30% of all incidents reported in hospitals in various countries.^[3–5]^ Despite efforts to keep patients safe, the incidence of inpatient falls in China was 18%. Falls may result in physical injury and increase the length of hospital stays and costs, leading to decreased activities of daily living; they may even be fatal, increase patient mortality and morbidity, and reduce the quality of life. Falls are common adverse events among individual inpatients owing to physical damage^[6]^; they may bring anxiety to patients and their families. Among patients suffering from falls, 30% to 70% develop depression syndrome.^[7]^

The present study aimed to identify the factors associated with inpatient falls. It conducted a retrospective analysis of the clinical characteristics and causes of falls, and it formulated nursing management countermeasures.^[8]^ This will help describe a profile of inpatients who fell at our facility and guide possible future interventions to decrease inpatient falls.

## 2 Methods

### 2.1 Participants

Our hospital received almost 175,200 inpatients in all wards from January 2019 to December 2020 and received 48,300 inpatients from March 2021 to October 2021. We conducted a retrospective analysis of fall incidents using the hospital database and non-punitive reporting system for adverse events. That system covers clinical errors or events that are submitted as incident or event reports regardless of whether or not they caused harm to patients.

We analyzed the medical records of 76 patients who had suffered falls in an inpatient unit of our hospital from January 2019 to December 2020. At the same time, the fall prevention group standard was established in early 2021 to intervene with patients. After the intervention, we retrospectively analyzed the medical records of 13 patients who had suffered falls in an inpatient unit of our hospital from March 2021 to October 2021, and compared them with those before the intervention. The inclusion criteria were fall events, including falls as defined by nursing adverse event management. We excluded outpatients, such as those attending the general outpatient clinic and digestive endoscopy center. Fall reports from inpatient units were reviewed preintervention (January 1, 2019–December 31, 2020) and postintervention (March 1, 2021–October 15, 2021). We compared demographic data, fall history, and Morse score variables before and after intervention.

This was a retrospective observational study of medical records; the data collected did not contain any details allowing identification of the subjects. This study met the requirements of the Declaration of Helsinki, and it was approved by the ethics committee of the Affiliated Hospital of Nantong University (Ethic: 2019-K060).

### 2.2 Process

We screened events that met our criteria, and event-related information was exported through the nursing adverse event reporting system at the Affiliated Hospital of Nantong University. The data included the following details: patient demographics; nurses on duty; incident overview; history of falls and risk factors; incident outcome; and continuous quality improvement records. We supplemented any missing information by accessing the case number. Nurse specialists graded the degree of injury caused by the falls according to the National Database of Nursing Quality Indicators: grade 0 (no injury); grade 1 (mild injury, such as bruising, swelling, a little bleeding, and pain); grade 2 (moderate injury and no permanent damage even with such results as excessive blood loss, lacerations, stitches, and splints); grade 3 (severe injury, such as fractures, nerve or internal injury, and cardiac arrest; this may require surgery, plaster casts, traction, and even result in death).^[10]^

We assessed fall risks by used specific tools in the prevention of falls. Morse Fall Scale was used to evaluate the risk of falls. In the present study, it was prioritized by the application of the Morse Scale, since it is a worldwide tool that classifies the risk of falling hospitalized in an effective way. This scale was translated and adapted transculturally into the Chinese, proving its great feasibility of application in the China reality. Each criterion evaluated receives a score that varies from 0 to 30 points, totalizing a risk score, whose classification is: low risk, from 0 to 24; moderate risk, 25 to 44, and high risk, ≥45. We obtained demographic data (incidence of falls, sex, and age), fall risk score, time of fall occurrence, location of occurrence, and adverse events (injuries requiring treatment) from the hospital database.

After a fall occurred, the nurse involved filled in the event overview in the nursing management system. The head nurse discussed the cause of the incident with related staff and submitted a report to the nursing department. The quality control department verified the report, reviewed it, and made its assessment of the cause of the incident; subsequently, that department supervised ongoing quality improvement. The same department implemented corrective measures and recorded the outcomes.

The inpatient fall incidence rate and fall injury grade ratio were computed according to the following equations: inpatient fall incidence rate (‰) = occurrence of inpatient falls per unit time/actual number of beds occupied by patients per unit time × 1000‰.

Inpatient fall injury grade ratio (%) = number of fall injuries per unit time in inpatients/number of inpatient fall cases within the period  × 100%.

### 2.3 Intervention

**Figure 1:**
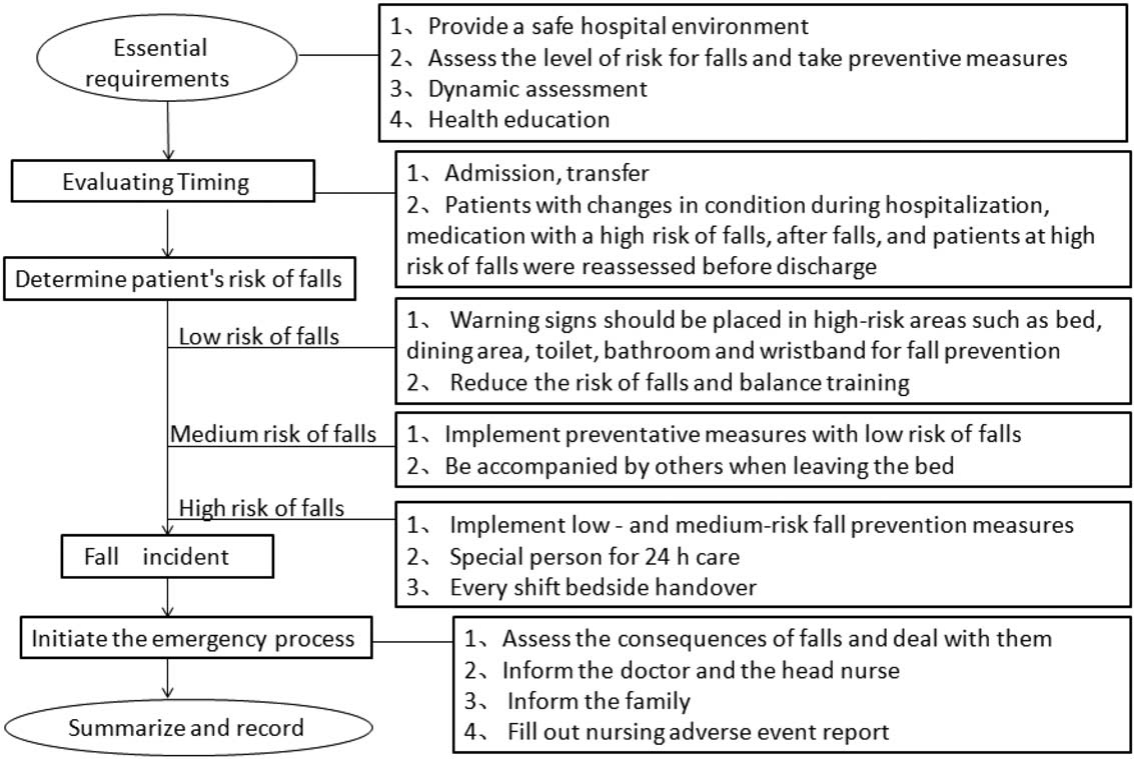
Fall prevention and management procedures.

The nursing department of Affiliated Hospital of Nantong University formed a fall prevention team. The chief of medicine was the head of the fall prevention group. The head nurse of the high-risk department, and a number of physicians at the university were selected as members of the fall prevention team. This effort underlined the importance of health education, process supervision, and enhancing patient participation in safety management. The aim was to find a new balance between patient functional exercise and safety management. In early 2021, our team revised the fall-prevention group stander (Table 1). At the same time, we formulated fall prevention and treatment procedures (Fig. 1). In addition to those procedures, the fall prevention team also developed nursing management countermeasures to prevention inpatient falls. This revised fall group standard was implemented in March 2021. Uniform training was provided to all nurses and patient care technicians.

**Table 1:** Preventive measures for fall-risk factors.

Dizziness and vertigo	Nurses should inform patients and caregivers in advance about dizziness and the possibility of falls caused by dizziness. Patients should be encouraged to keep a diary about their headaches and vertigo. Nurses should evaluate patients sensation of dizziness and vertigo as well as predisposing factors, duration and intensity, nature, associated symptoms, and relieving methods. Patients should be told that if they feel dizzy, they should squat or lean on a firm, stable object. Patients and caregivers should be encouraged to attend vestibular therapy administered by rehabilitation physicians.
Visual impairment	If patients use >2 pairs of glasses for different purposes, appropriate labels should be affixed. Nurses should instruct patients with a history of falling or at risk of falls owing to visual impairment to use single pair of glasses. When nursing a patient with hemianopia, it is advisable to stand on the blind side, and enhance the patient's perception of space and location through sound. If a patient has undiagnosed vision problems, nurses should report that to the physician.
Abnormal muscle strength, balance, and gait	Nurses should observe patients and ask them questions about walking and balance. Patients should be encouraged to participate in muscle strength, balance, and gait training programs developed by rehabilitation physicians; implementation should be supervised. Patients should be instructed in the proper use of protective devices, such as walking aids. Patients suffering from severe osteoporosis and hip fracture should receive assistance in wearing hip protectors.
Postural hypotension	Patients should be instructed to change their posture slowly, avoid standing up suddenly after bending, and reduce the frequency and degree of bending. When instructing the patient to change from a lying to a standing posture, a three-part approach should be adopted: lying flat for 30 s; sitting for 30 s; standing for 30 s, and then walking. It is advisable to guide patients to shower at 37–40 °C. It is necessary to inform patients about aerobic endurance training, intermittent tiptoe standing, and alternating weight bearing with both lower limbs. If orthostatic hypotension occurs, patients should sit down immediately or lie flat to rest.
Incontinence and frequent excretion	It is advisable to place the patient close to a toilet or provide alternative facilities for washing and toileting at the bedside. The causes of incontinence should be observed and identified. Nurses can train patients to control their discharge of urine and feces. It is recommended that patients follow a toilet plan. Patients who use the toilet frequently should use such items as large or urine incontinence care pants, nursing beds.
Use medications with a high risk of falls	Nurse should clearly inform patients and caregivers of medications that could increase the risk of falls. If patients take medications that have a high risk of falls, they should limit their activities while under such treatment.

### 2.4 Statistical analysis

We conducted all statistical analyses using IBM SPSS, version 23.0 (SPSS Inc. Chicago, IL). We applied *P* < .05 for statistically significant differences. The characteristics were described as means ± standard deviations, frequency and percentage or composition ratios, and incidence. Two sets of continuous variables were measured using *t* tests or nonparametric rates using chi-squared or something like that. We analyzed the clinical characteristics related to inpatient falls.

## 3 Results

**Figure 2:**
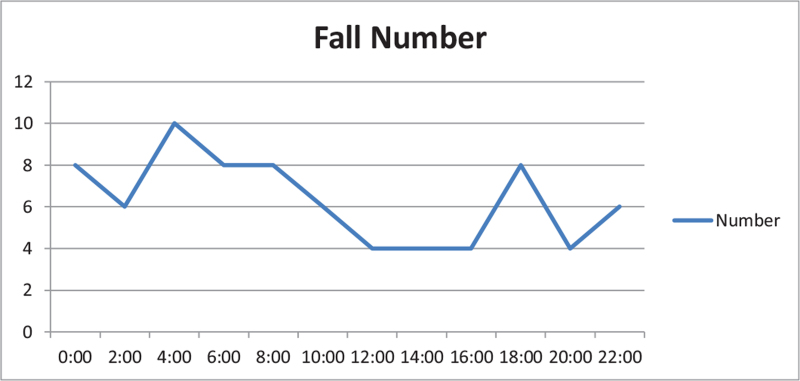
Time distribution of fall occurrence in 76 inpatients.

Before intervention, the fall rate was 0.0434% (76/175,200). The most common occurrence time was during the night shift (18:00–08:00). The distribution of falls at different times of day is shown in Fig. 2.

The patients fell more frequently in the hospital ward (97.3%), toilets (34.2%), and at their bedsides (42.1%), and the most common type was collapse (68.4%). Notably, 39.5% of the patients were unaccompanied at the time.

Regarding the consequence of the falls, 18 patients (23.6%) suffered no injury, 22 (28.9%) had mild injury, 12 (15.8%) had moderate injury, and 24 (31.5%) had severe injury. Most cases (52.6%) occurred in the internal medicine department (Table 2).

**Table 2:** Characteristics of the fall events (n = 76).

Variables	Categories	n	Composition ratio (%)
Place	Ward	74	97.3
	Bedside	32	42.1
	Toilet/bathroom	28	36.8
	Corridor	8	15.8
	Examination room	2	2.6
	Outside ward	2	2.6
Activity	Changing position	32	42.1
	Walking	8	15.8
	Standing	14	18.4
	Sitting	18	23.7
Fall type	Collapse	52	68.4
	Slipping	6	7.9
	Tripping down	4	5.3
	Being knocked	6	7.9
	Falling from bed	8	10.5
Accompanied by	Family	26	34.2
	Caregiver	20	26.3
	No one	30	39.5
Main injury	None	18	23.7
	Mild	22	28.9
	Moderate	12	15.8
	Severe	24	31.6
Department	Internal	40	52.6
	Surgical	20	26.3
	Other	16	21.1

The primary causes of falls were categorized in the ongoing quality improvement record for events. As shown in Table 3, the top 3 causes of the falls accounted for 84.2% of cases; of those, 30 cases (39.5%) were caused by caregivers, 20 (26.3%) by the patients themselves, and 14 (39.5%) by nurses.

**Table 3:** Classification of causes of falls among the 76 inpatients.

Classification	Reason	n	Percentage (%)
Caregiver factors	Accompanying personnel or family members left the patient without handing over to the nurse; accompanying care was inefficient.	30	39.5
Patient factors	Patients had strong autonomous awareness, overestimated their personal ability, refused being accompanied, nurse assistance, or felt reluctant to trouble their families; their compliance was poor.	20	26.3
Nurse factors	The nurse did not accurately assess the fall risk and did not accurately predict high risk; safety education and protection measures were not appropriately implemented; the nurse did not consider the patient's observations and demands in a timely manner.	14	18.4
Environmental facility factors	The beds were too high; the floor was slippery; the light was dim; the wheelchair, toilet, or bathroom facilities were not skid resistant.	4	5.3
Disease factors	The patient's illness led to inevitable falls: advanced cancer, hypoglycemic medication, sedatives, laxatives, and other special drugs.	8	10.5

After intervention, the fall rate was 0.027% (13/48,300). This difference was statistically significant (*P* = .023). After implementing the fall prevention standard, the fall rate decreased significantly. The risk of fall and fall injury were significantly reduced. Before and after intervention, there was no statistical difference in age, marriage, sex, and other sociological demographic data of patients who fell. The summary of these results appear in Table 4.

**Table 4:** Summary of falls patient exploration for preintervention/postintervention analysis.

Characteristics	Preintervention (n = 76)	Postintervention (n = 13)	*P*
Age, y	68 (62, 75)	68 (57.5, 79)	.706
Gender			.343
Male	49 (64.5)	6 (46.2)	
Female	27 (35.5)	7 (53.8)	
Education, y			.783
≤9 y	36 (47.4)	9 (11.8)	
>9 y	40 (52.6)	4 (30.7)	
Independent living			.105
Yes	4 (5.3)	5 (38.7)	
No	72 (94.8)	8 (61.5)	
Fall history			.085
Yes	33 (43.4)	9 (69.2)	
No	43 (56.6)	4 (30.8)	
Fall risk			.010
Low risk	29 (38.2)	8 (61.5)	
Middle-high risk	47 (61.8)	4 (30.8)	
Morse score	35 (15, 35)	20 (5, 32.5)	.140
Injury type			.024
0	18 (23.7)	7 (53.9)	
1	22 (28.9)	4 (30.8)	
2	12 (15.8)	2 (15.4)	
3	24 (31.6)	0 (0)	
Fall factors			.977
Caregiver factors	30 (39.5)	3 (23.1)	
Patient factors	20 (26.3)	4 (30.8)	
Nurse factors	14 (18.4)	1 (7.7)	
Environmental facility factors	4 (5.3)	2 (15.4)	
Disease factors	8 (10.5)	3 (23.1)	

## 4 Discussion

This study found that inpatients falls were characterized by a high incidence of falls, multiple peaks in the distribution of the time of day, and a wide distribution of hospital locations.^[9]^ We observed a large proportion of moderate and severe fall injuries.

Falls are a common health problem among inpatients; in the adult population, the incidence of falls increases with age.^[10,11]^ We found that among the 89 fall cases, the average age was 68, indicating a clear aging trend. Our findings are consistent with those of previous studies. Older inpatients are at a higher fall risk owing to such factors as aging, illness, treatment, and passive adaptation to an unfamiliar hospitalization environment. Injurious falls can lead to high disability and mortality, resulting in prolonged hospital stays and increased medical expenses.

The most common time for the patients to suffer a fall was from 05:00 to 07:00.^[12]^ That could be because they got up early and moved without being completely awake. At that period, Chinese nurses are typically busy collecting blood samples and doing their morning duties: they may have failed to anticipate patient risk or attend to their needs in time. This finding is consistent with that of Chen Yumei. We observed a jagged distribution of falls at other times of day, and small peaks occurred at multiple time points. This suggest that nursing staff should enhance their inspections of patients (especially older ones) in the early morning as well as in the evening when patients visit the toilet, at nap up, when they shower, and at other times. In that way, staff could meet the needs of older patients and eliminate the risk of falling.

We found that falls occurred most frequently at the bedside (42.1%) and in the toilet and bathroom (36.8%); 18.4% were in other locations outside the ward, such as the corridor and examination room. We observed that most falls were caused by caregivers failing to implement safety measures. According to one report, 10% to 25% of falls were due to muscle weakness.^[13]^ Studies have shown that 10% of people aged over 65 years need assistance to cross a room, 20% require assistance to climb stairs, and 40% need assistance to walk up to 500 m.^[14]^ Public hospitals in China typically extend over many buildings; accordingly, patients have to go to various places for examinations and treatment. Preventing falls among elderly patients is closely related to the people who accompany them: caregivers should attend to older patients as much as possible. However, the present study found that the caregivers had failed to meet their responsibilities in this regard.

In the present group of patients, falls led to different degrees of injury: serious injuries of 76 falls accounted for 31.6%, which is higher than the figure reported in previous studies. With increasing age, the risk of falling among older people rises annually; 50% of falls lead to serious injury, which seriously affects the health and quality of life of older people.^[15]^ Studies have shown that standardized fall-prevention training can reduce the incidence of falls and ensure patient safety.^[16]^ Assessing fall-risk factors is particularly important for predicting and preventing falls in older people.^[17]^ Accordingly, it is necessary to prevent falls among older patients. The most effective way to prevent hospital falls is multifactor assessment and intervention, which can reduce the number of falls by 20% to 30%.^[18]^ Our hospital applied a Morse tool to assess inpatient fall risk, it was possible to identify various associated factors (including medical and social conditions), thereby leading to effective fall-prevention programs.

Our analysis of the primary causes of falls recorded in the fall event report revealed that the top 3 causes among the 76 inpatients accounted for 84.2%: inappropriate management by nursing staff in preventing falls; insufficient participation among the patients in fall-prevention management; and poor patient accompaniment by caregivers. The adverse nursing events we identified were reported through the nursing management platform network: a nonpunitive nursing culture has developed over many years in China.^[19]^ The nursing department at our hospital made reports about nursing adverse events for the general benefit. In that way, the department has clarified matters such that caregivers fully understand that prevention is the goal of adverse reporting. Thus, the causes of inpatient falls recorded in the ongoing quality improvement records identified in the present study were discussed by the nursing department, quality control department, and fall team toward reducing reoccurrence. It has been shown that identifying factors is key to preventing falls.^[20]^

Managing inpatient fall prevention includes at least the following: accurate and comprehensive nursing evaluation; identifying high-risk falls; effective implementation of measures for fall prevention; health education for patients and their caregivers; evaluating the effect of health education; and formulating targeted emergency plans. This present study found that in addition to the reasons frequently mentioned in previous research (such as inaccurate nurse assessment and inadequate inspection), inadequate implementation by nurses of safety education and lack of supervision when undertaking preventive measures are important causes of older patients suffering falls. In clinical nursing, falls are caused by such factors as patients having a strong sense of autonomy, poor compliance, and overestimating their own abilities. These are common problems and constitute a difficulty in efforts to prevent patient falls.

In the present study, the causes of the 76 inpatients having suffered falls were discussed by the nursing department, and those findings were then reviewed by the quality control department. A frequently identified characteristic was patients having a strong sense of autonomy. Such expressions as “stubborn character,” “defiance among older patients,” “deliberately concealing their personal history of falling,” “a tense relationship with the family,” and “refusal to be accompanied in the hospital” appeared repeatedly in the descriptions of the incidents. That finding reflects the patients’ low degree of participation and poor cooperation in safety management.

In addition to the nurse and patient factors, we identified caregiver factors and environmental facilities. Other factors included high beds, slippery floors, dim lighting, and non-slip facilities for wheelchairs and in toilets and bathrooms.

The characteristics and causes of the falls were analyzed in depth. It was evident that the falls occurred more frequently at night and at certain times. Patients receiving particular kinds of medication at night were more prone to suffer falls. However, patients also fell more frequently when using the toilet. We formulated fall-prevention and treatment procedures. In addition to those procedures, the fall-prevention team also developed nursing management countermeasures to prevention inpatient falls.

Our results demonstrating fewer falls per patient day after implementing the patient fall-prevention stander were similar to those of the Health Research and Educational Trust study.

Our study using the regarding fall-risk assessment, multivariate assessment, and intervention are the most effective measures to prevent and reduce the number of hospital falls.^[21]^ We determined the risk for adults using a fall-assessment score based on Morse. We referred to the assessment form known as the Humpty Dumpty Scale (an overseas tool for assessing children's fall risk) to determine our patients’ fall risk.

With respect to effective interventions for falls, corresponding preventive measures should be taken according to different fall-risk levels. Through fall-risk assessment, interventions can correct and manage the various risk factors. It is often necessary to adjust patient medications, address hazardous environments, and apply fall-prevention techniques, such as mobile alarms, hip protection, and ultra-low beds. At Nantong University, we also provide appropriate preventive measures for fall-risk factors. Our working group has applied several procedures for fall prevention. Those measures include the following: frequent evaluation using the fall-assessment score sheet; enhancing awareness about falls among patients and their families on admission; and providing information on medication use, including sleeping pills and psychotropic drugs. Patients at a high risk of falls are usually placed in a ward close to the nurses’ station; a monitoring system is employed by means of a sensor device. The results of the present study suggest that special care is necessary for such patients. We currently use a wristband whose color indicates whether there is a high degree of risk for falls; that is recognized by all medical staff when they come into contact with the patients. At our university, inpatients do not wear slippers because they lack heels: fall prevention is promoted by wearing shoes with a heel, such as athletic and rehabilitation shoes.

It is necessary to inform patients about the benefits of health education effects and process supervision: that helps encourage their own participation in safety management. Conventionally, health education is mainly based on oral explanations given to patients by doctors and nurses. In recent years, with the expansion of information technology, it is possible to provide various forms of health education using network platforms. To educate our patients about the dangers of falls, we have produced a video concerning the complications following falls, and it is broadcast within our hospital. We have also conducted health education for patients using feedback teaching, which is widely applied in medicine overseas.^[22]^ With that teaching, our nurses repeatedly evaluate patients and clarify the current medical situation to them; the nurses also assess the extent to which the patients understand the content. This process enriches the communication between nurses and patients. In addition, working group activities for fall prevention are important—particularly for patients at high risk of falls. Educating patients and ward staff as part of routine clinical practice could reduce the incidence of falls and related injuries: it should therefore be incorporated into routine fall-prevention programs.

We use the fall prevention standards and management countermeasures. According to the standards, based on Morse, patients can be effectively assessed, and targeted nursing intervention can be given according to the risk level. The impact of fall incidence and fall injury is reduced, and the intervention is effective.

## 5 Conclusion

This study has the following limitations. We examined patients in: all wards rather than focusing just on patients with certain diseases or in a specific department. All the fall cases were reported using the nursing adverse event management system. The investigation sample after intervention is small and the study time is short. However in light of these limitations, we analyzed the fall events and conducted risk management for them. Following our analysis of the falls, we developed a series of preventive measures and procedures. After intervention, the occurrence of fall, fall injury, and fall risk were significantly reduced. The results of this study should be conductive to preventing falls and associated adverse events.

## Acknowledgments

The authors thank Liwen Bianji (Edanz) (www.liwenbianji.cn/ac), for editing the language of a draft of this manuscript.

## Author contributions

**Conceptualization:** Xiaoling Zhu.

**Data curation:** Xiaoyan Liu.

**Investigation:** Xiaoyan Liu, Xiaoling Zhu.

**Methodology:** Xiaoling Zhu, Yan Song.

**Resources:** Xiaoyan Liu.

**Supervision:** Xiaoling Zhu.

**Validation:** Yan Song.

**Visualization:** Yan Song.

**Writing – original draft:** Xiaoyan Liu.

**Writing – review & editing:** Yan Song and Xiaoyan Liu
